# Experimental evolution and genome sequencing reveal variation in levels of clonal interference in large populations of bacteriophage φX174

**DOI:** 10.1186/1471-2148-8-85

**Published:** 2008-03-17

**Authors:** Kim M Pepin, Holly A Wichman

**Affiliations:** 1 Department of Biological Sciences, University of Idaho, Moscow, ID, 83844-3051, USA; 2 Center for Infectious Disease Dynamics, Penn State University, University Park, PA, 16802, USA

## Abstract

**Background:**

In large asexual populations where beneficial mutations may co-occur and recombination is absent, the fate of beneficial mutations can be significantly affected by competition (i.e., clonal interference). Theoretical models predict that clonal interference (CI) can slow adaptation, alter the distribution of fixed beneficial mutations, and affect disease progression by impacting within-host evolution of pathogens. While phenotypic data support that CI is a significant determinant of adaptive outcome, genetic data are needed to verify the patterns and to inform evolutionary models. We adapted replicate populations of the bacteriophage φX174 under two levels of CaCl_2 _to create benign and harsh environments. Genomic sequences of multiple individuals from evolved populations were used to detect competing beneficial mutations.

**Results:**

There were several competing genotypes in most of the populations where CaCl_2 _was abundant, but no evidence of CI where CaCl_2 _was scarce, even though rates of adaptation and population sizes among the treatments were similar. The sequence data revealed that observed mutations were limited to a single portion of one gene in the harsh treatment, but spanned a different and larger region of the genome under the benign treatments, suggesting that there were more adaptive solutions to the benign treatment.

**Conclusion:**

Beneficial mutations with relatively large selection coefficients can be excluded by CI. CI may commonly determine the fate of beneficial mutations in large microbial populations, but its occurrence depends on selective conditions. CI was more frequent in benign selective conditions possibly due to a greater number of adaptive targets under this treatment. Additionally, the genomic sequence data showed that selection can target different types and numbers of phenotypes in environments that differ by only a single continuous variable.

## Background

In asexual populations, adaptive evolution is fueled by novel beneficial mutations that arise and sequentially fix due to selection. However, not all of these beneficial mutations may reach fixation; when they co-occur, competition for fixation ensues [1,2]. Those with higher selection coefficients are more likely to win the competition resulting in loss of the smaller effect mutations, a phenomenon called 'clonal interference' [3,4]. A less severe version of this type of interference, that can decrease the efficacy of selection in sexual organisms, occurs when a locus under weak selection is tightly linked to a more strongly selected locus (i.e., Hill-Robertson Effect; [5,6]). Both levels of interference can significantly impact adaptive outcome by decreasing the rate of adaptation, altering levels of standing genetic variation, and favoring the evolution of sex [3,7-13]. A better understanding of the effects of interference on the fate of beneficial mutations is not only important in advancing our knowledge of evolutionary processes; it also has great practical relevance in monitoring and predicting disease progression in hosts with chronic infections [14].

Theoretical studies of adaptation in large populations of asexual organisms show that the strength of interference is positively correlated with population size and mutation rate because mutations have a higher probability of co-occurring under these conditions [3,12,13,15,16]. Under the assumption that the number of available beneficial mutations is infinite, CI is predicted to: 1) slow the rate of adaptation by delaying fixation of beneficial mutations, and 2) result in a larger mean selection coefficient of fixed beneficial mutations (i.e., mutations with higher selection coefficients out-compete smaller ones). Kim and Orr [17] tested whether fixation of beneficial mutations is delayed when only a finite number of beneficial mutations are available in a given genome. Their study showed that CI can have little effect on the speed of adaptation in large populations when few beneficial mutations are available, or when the distribution of beneficial mutations has a high mean and variance. When beneficial mutations are extremely rare, each mutation may reach fixation before a competitor arises. Likewise, when selection coefficients are very different, the transition time (i.e., time it takes a beneficial mutation to fix once it has occurred) may be so short that the large effect mutation reaches fixation before competitors arise. Ultimately, it is the interplay of these factors, mutation number and transition time, that determines the level of CI. Thus, our understanding of adaptation in large asexual populations could be improved by examining how parameters of the distribution of selection coefficients for beneficial mutations (i.e., overall number, mean, and variance) influence levels of CI. Manipulating environmental conditions is a feasible experimental approach for testing effects of the distribution of beneficial mutations on levels of CI.

A more recent study of adaptation in large asexual populations distinguishes between a one-by-one fixation regime under clonal interference and a multiple mutation regime where competing genotypes may have more than one mutation [9]. In contrast to the one-by-one model of clonal interference, which predicts decelerated adaptation rates and transient levels of genetic variation at large population size (N) and high beneficial mutation rate (μ_b_), the multiple mutation model predicts that the both the speed of adaptation and variation in fitness increase logarithmically with N and with μ_b_.

Consistent with predictions from the one-by-one clonal interference models [3,12,15,16], experimental evolution studies with *E. coli *and vesicular stomatitis virus have shown that adaptation rates plateau, and that higher population fitness is achieved as population sizes and mutation rates increase [4,18-20]. However, since these data are phenotypic, CI is not the only explanation for the observed patterns. In larger populations, adaptive mutations will have longer transition times even in the absence of competitors, and there may be few adaptive mutations available under some conditions, especially if the first few mutations that fix leave the population stranded at a low local fitness peak. More recent studies have provided a compelling demonstration that competition between beneficial mutations affects the probability that genotypes are lost, emphasizing the importance of multiple simultaneous mutations in determining adaptive trajectories [21,22]. Yet another recent study of adaptation in large yeast populations did not observe that the speed of adaptation decreased during adaption under conditions where multiple mutations were presumed to be pervasive, and found that variation in fitness was higher at higher Nμ_b_, supporting the multiple mutation model of adaptation [9]. These more recent studies have also tested predictions of the underlying models of adaptation by observing patterns of phenotypic adaptation. None of the empirical studies have investigated the presence of competing mutations with genomic sequence data, or examined the number of genotypes that may be competing at any given time, although Holder and Bull [23] did observe high frequency polymorphisms during experimental evolution of bacteriophage in the family Microviridae. Examining the underlying genetics of CI in large populations evolved in different environments would increase our mechanistic understanding of adaptation in large microbial populations and identify the importance of considering multiple concurrent mutations in models of adaptation.

A direct way to identify CI in experimental evolution is through genome sequence analysis. Beneficial mutations must be present simultaneously for CI to affect their fixation. Populations with mutually exclusive adaptive genotypes that coexist at relatively high frequencies indicate CI. The bacteriophage φX174 is useful for identifying CI through sequence analysis and experimental evolution because it is small enough that genomic sequence data can be obtained for multiple individuals at multiple time points during the evolutionary trajectory [23-29]. Because each phage produces about 100 infectious progeny per 20 minutes, growing populations must be diluted frequently during long-term propagation. The bottleneck effects from flask-to-flask transfers are so strong during experimental evolution of φX174 that there is an extremely low probability that small-effect or neutral mutations will attain appreciable frequency in the population. When only a small number of individuals are sampled from a large phage population, there is likewise an extremely low probability of sampling an evolved genotype two or more times unless it has ascended to high frequency. The presence of multiple high frequency genotypes in a population indicates either a selective sweep in progress or CI, depending on the nature of the differences between genotypes.

To identify the underlying genetics of CI we evolved large populations of φX174 and sequenced genomes of isolated genotypes from mid- and endpoint populations. We evolved similarly large populations of phage under different levels of CaCl_2 _to create benign versus harsh conditions in order to test effects of environment on the frequency of CI. We expected that differences in CaCl_2 _abundance would alter parameters of the distribution of available beneficial mutations, thus influencing levels of CI. We assumed that by limiting the same resource to different degrees, selection would target the same trait(s) in each treatment, but that mutations that could potentially improve the trait(s) would be proportionately more beneficial in harsher resource conditions. In other words, we expected that the number of available beneficial mutations (waiting time) would be similar among treatments, but that the mean selection coefficient of beneficial mutations would be higher in the harsh environment since these populations would be further from their local fitness optimum. Under these assumptions, we predicted that CI would be more prevalent under higher resource levels since the smaller-effect mutations would have longer transition times, allowing for their co-occurrence. In contrast to our assumptions, we did not find evidence of stronger selection in the harsh environment and did find evidence that there may have been fewer beneficial mutations available in the harsh environment. Thus, although our resource treatments did not have the effects we expected, they did alter the distribution of available beneficial mutations in a manner that yielded much needed insight into the effects of environment on the frequency of CI.

## Results

### Pre-adaptation to control conditions

Growth rate of the lab strain increased dramatically (by 5.5 ± 0.8 population doublings/hr) during the first 35 passages in experimental conditions, and very little (0.7 ± 0.6 population doublings/hr) during the next 21. At this point (56 transfers, 27 hours of growth), we sequenced an isolate and found three novel mutations (C878T, G2085C, and C2179T). We then continued the adaptation for 49 more transfers (52 hours of growth) and sequenced another plaque isolate. No additional mutations were found, and thus we concluded that the genotype was fully adapted to the control conditions such that it would not be expected to undergo further adaptive evolution under control conditions. We measured fitness of this triple mutant at different concentrations of CaCl_2 _to determine conditions where fitness was significantly decreased relative to those used in the pre-adaptation. We used this genotype as the ancestor (anc) to initiate the first line in each treatment. When the control line reached the midpoint of the evolutionary trajectory, we sequenced 10 isolated genotypes and found that all 10 had an additional mutation, C788T. In an effort to maintain a control line, we used this genotype (anc^788^) to initiate the second and third lines in each treatment before we knew that other adaptive mutations would be detected in the endpoint population of the first control line. Thus, the second and third lines are true replicates of each other, while the first line in each treatment was initiated from a different ancestor.

Additionally, since the control lines behaved like the benign treatment, they were treated as such in the analyses.

### Adaptation to treatments

**Figure 1:**
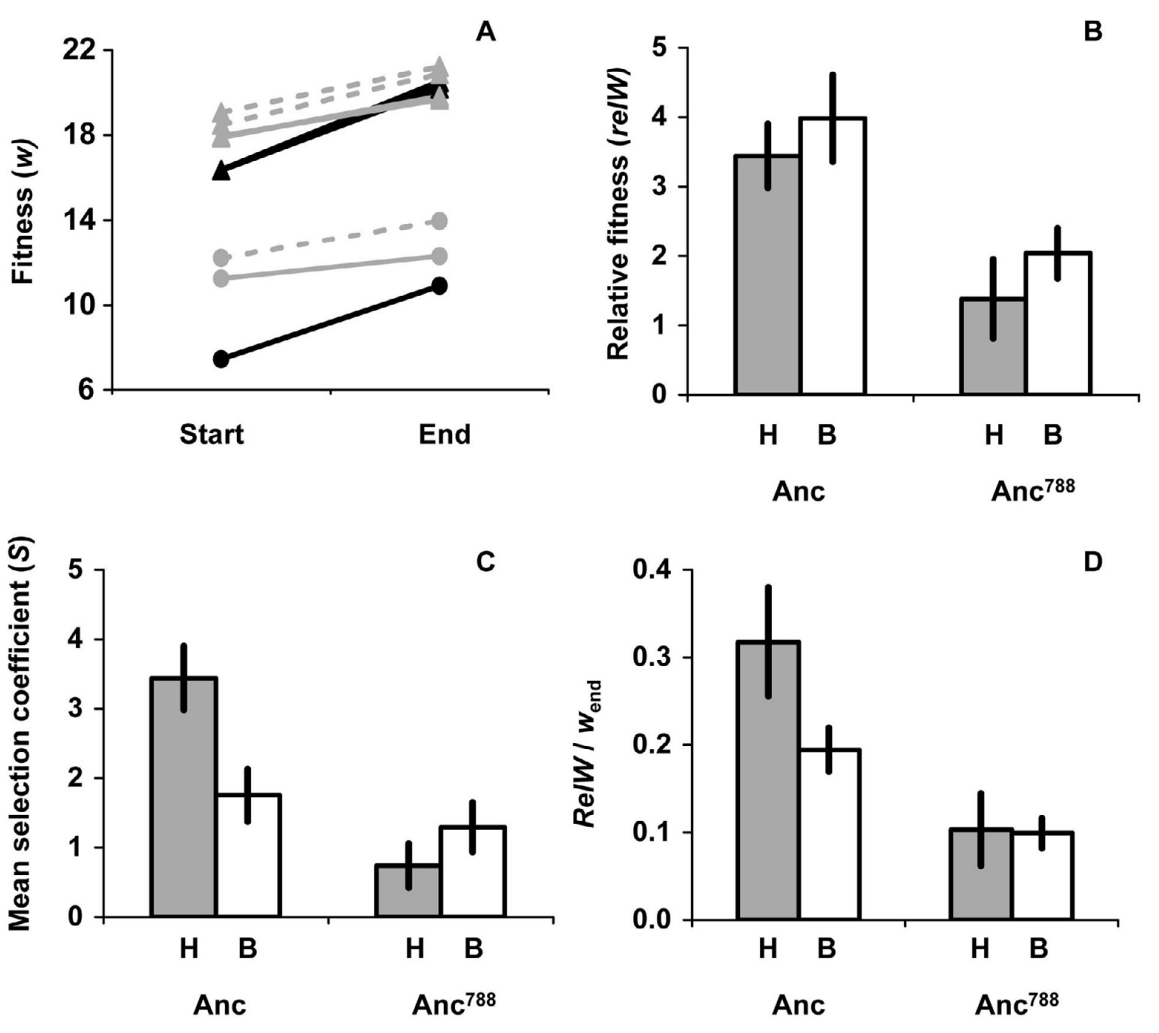
**Adaptation and selection strength**. (A) Change in fitness for each experimental line. Data are means for five replicate, paired fitness assays of the ancestor and the endpoint population of each line in its respective treatment. anc: black lines (all line 1), anc^788^: grey lines, solid grey: line 2, dashed grey: line 3, circles: harsh, triangles: benign. (B) Fitness of each ancestor relative to endpoint populations (n = 5). Data from benign environments are pooled, as are data from lines 2 and 3 for anc^788 ^(within the H and B treatments). Bars are 95% confidence intervals from t-tests of mean differences between the harsh (H) and benign (B) treatments for each ancestor. (C) Mean selection coefficient of mutations in endpoint populations (n = 5). Data from benign environments are pooled, as are data from lines 2 and 3 for anc^788 ^(within the H and B treatments). Bars are 95% confidence intervals from t-tests of mean differences between the harsh (H) and benign (B) treatments for each ancestor. (D) The change in fitness as a proportion of total fitness (n = 5). Data from benign environments are pooled, as are data from lines 2 and 3 for anc^788 ^(within the H and B treatments). Bars are 95% confidence intervals from t-tests of mean differences between the harsh (H) and benign (B) treatments for each ancestor.

To examine potential differences in adaptation among the treatments with different ancestors we first conducted a two-factor ANOVA with interaction on the relative fitness data, where ancestor and treatment were the main effects. There was a significant difference in adaptation between the two ancestors (*F*_1,43 _= 63.2, *P *< 0.0001; Fig. 1A), but no significant effect of treatment (*F*_2,43 _= 2.7, *P *< 0.083) or the interaction of ancestor and treatment (*F*_2,43 _= 0.38, *P *< 0.69), indicating that fitness increased by similar amounts among the resource treatments. Since the control and benign environments were very similar treatments in terms of both the level of CaCl_2 _and the starting fitnesses of the ancestor (Table 1), we pooled these two treatments to increase power for testing differences in adaptation between the benign and harsh environments. Adaptation was not significantly different between the benign and harsh treatments for anc (*t*_13 _= 1.8, *P *< 0.10). On the other hand, adaptation of anc^788 ^was marginally different between the two environments (*t*_17 _= 2.1, *P *< 0.053). This difference was statistically significant if we analyzed the data by a nested ANOVA that accounted for potential variation due to the different lines within treatments (Treatment: *F*_1,28 _= 4.6, *P *< 0.043; Line nested in treatment: *F*_4,28 _= 1.0, *P *< 0.41). Overall, the data showed that there was no significant difference in the amount of adaptation between the benign and harsh environments for anc, but that the amount of adaptation may have been slightly higher in the benign environment for anc^788 ^(Fig. 1B). Due to these differences and the finding that there was no significant variation from lines within treatments, we analyzed fitness data from each ancestor separately, but pooled data for the benign treatments, and data for anc^788 ^lines within each benign and harsh treatment for subsequent analyses of adaptation.

**Table 1:** Parameters of Experimental Evolutions.

		Per flask: (means)									
Treatments anc	Line	***N***_*o*_	***N***_30_	**N**_*o*_**/N**_30_	Total hrs	Total flasks	***w***_*o*_	***w***_*e*_	*relW*	*S*	***relW/w***_*e*_
Control	1	1.8 × 10^6^	5.7 × 10^9^	0.0003	74.8	147	16.3	20.5	4.1	1.4	0.20
Benign	1	1.6 × 10^6^	6.4 × 10^9^	0.0003	70.8	138	16.3	20.2	3.9	2.1	0.19
**Harsh**	**1**	**1.0 × 10**^7^	**5.7 × 10**^9^	**0.0014**	**61.4**	**66**	**7.5**	**10.9**	**3.4**	**3.4**	**0.32**
**anc**^788^											
Control	2	2.3 × 10^6^	3.5 × 10^9^	0.0006	87.4	174	18.0	19.7	1.7	0.8	0.086
	3	1.3 × 10^6^	4.8 × 10^9^	0.0003	63.5	126	19.1	21.2	2.2	1.1	0.10
Benign	2	3.1 × 10^6^	7.9 × 10^9^	0.0004	38.4	76	17.9	19.8	1.9	1.9	0.097
	3	3.2 × 10^6^	7.5 × 10^9^	0.0004	43	85	18.5	20.9	2.4	1.3	0.11
**Harsh**	**2**	**2.4 × 10**^7^	**4.5 × 10**^9^	**0.005**	**68.5**	**119**	**11.3**	**12.3**	**1.0**	**0.5**	**0.08**
	**3**	**6.3 × 10**^7^	**6.5 × 10**^9^	**0.01**	**48.3**	**80**	**12.2**	**14.0**	**1.8**	**1.0**	**0.12**

Treatment: Control = 2.0 mM CaCl_2_, Benign = 1.7 mM CaCl_2_, Harsh = 0.2 mM CaCl_2_.

*N*_*o *_and *N*_30_: Mean phage population sizes at the start and end of each flask growth period.

*N*_*o*_*/N*_30_: Bottleneck size; lower numbers indicate lines where beneficial mutations had higher extinction probabilities from bottlenecks.

*w*_*o*_: Mean fitness of the ancestor in treatment conditions (n = 5).

*w*_*e*_: Mean fitness of the endpoint populations in treatment conditions (n = 5).

*relW: *Mean fitness of the evolved population relative to ancestor (n = 5).

*S*: Mean selection coefficient; a measure of selection strength.

*relW*/*w*_*e*_: Fitness change as a proportion of total fitness; a measure of selection strength.

"Harsh" treatments are shown in bold.

### Selection strength

Selection strength is determined by parameters of the distribution of beneficial mutations. If the mean selection coefficient (*S*) is large, then selection is strong. Thus, to evaluate differences in selection strength between treatments, we first compared the average selection coefficient of mutations by dividing the relative fitness by the average number of mutations per genome, which was weighted by the frequency of genotypes with each particular number of mutations (see Methods). This measure takes into account both the frequency and effect of observed mutations to estimate the average selection coefficient, but makes the simplifying assumption that mutations have equal selection coefficients. We found that selection strength was significantly higher in the harsh environment for anc (*t*_11 _= 7.18, *P *< 0.0001; Fig. 1C), but that oppositely, selection strength was stronger in the benign environment for anc^788 ^(*t*_26 _= 2.52, *P *< 0.018; Fig. 1C). Another dimension of selection strength is the amount of fitness gain as a proportion of total fitness. For example, if two evolved populations each increase in fitness by 2, but one population has a total fitness of 4 and the other population has a total fitness of 8, then mutations occurring in the former case cause proportionately more fitness gain which could indicate stronger selection. Thus, as a second method of evaluating selection strength, we compared the proportional fitness gain between the two treatments for each ancestor. By this measure, selection strength was again stronger in the harsh environment for anc (*t*_6 _= 4.89, *P *< 0.0028; Fig. 1D), but was not different between the two treatments for anc^788 ^(*t*_13 _= -0.19, *P *< 0.86; Fig. 1D). Overall, there was evidence that selection was strongest for anc in the harsh environment. However, since only a single mutation was found for anc in the harsh environment, it is possible that we observed this pattern by chance, and thus we did not consider this a strong result.

### Nature of the genetic changes

#### a. Determining which mutations are adaptive

CI is a process concerning adaptive mutations, yet we did not measure the fitness effects for most of the individual mutations observed in this experiment. In order to identify populations undergoing CI, we used sequence data (Table 2) and evolutionary theory to infer that most, if not all, of the mutations were adaptive. We note that the bottleneck sizes in our experiment almost completely excluded mutations with selection coefficients less than 0.1 (Fig. 5 *in *[30]), suggesting that: 1) our design was biased towards sampling large-effect mutations, and 2) neutral mutations had negligible probabilities of rising to appreciable frequencies. However, in an effort to be conservative, we considered whether each mutation showed other evidence of being adaptive (Table 3). A common criterion for identifying adaptive mutations from sequence data is parallel evolution [24,26,31]. We found that ten of the 23 different mutations were observed in multiple lines in this study, and seven of these ten parallel mutations were observed in multiple isolates in at least one line, suggesting that they had attained high frequency. Of the two parallel mutations not observed at high frequency, one was observed six times in previous studies. Of the 14 mutations observed in only one line in this study, nine were observed in more than one isolate (i.e., high frequency). Although it is possible that a mutation could reach high frequency by hitchhiking with a large-effect beneficial mutation, the substitution dynamics for eight of these nine high frequency mutations indicate that hitchhiking does not explain their ascent to high frequency; hitchhiking could not be ruled out for G2973A. Thus, there is strong evidence that 18 of the 23 different mutations are adaptive. There are two mutations where evidence for their adaptive nature is not as strong – G2973A and C581T (present in one midpoint isolate). In the case of G2973A, this mutation has been observed previously under similar conditions (Pepin, K.M., unpublished data), and it is in a codon adjacent to mutation C2971T, which showed strong evidence of being adaptive in the current study. Likewise, the mutation C581T was observed previously under similar conditions (Pepin, K.M., unpublished data), and it results in a non-synonymous substitution that is two amino acids away from one that occurred independently in two lines of this experiment (protein E, site 7), as well as being two away from a site known to have a large effect on lysis regulation (protein E, site 3, [32]). There is no evidence that the remaining four of the 23 mutations were adaptive (Table 3). Interestingly, neither the adaptive nature of the two mutations where evidence is weaker, nor the adaptive nature of these four mutations, affects our conclusions regarding the presence of CI in any replicate.

**Table 2:** Genotypes Identified by Genome Sequencing.

Anc	Treatment	Line	^a^**Midpoint genotypes**	^b^**Total**	^a^**Endpoint genotypes**	^b^**Total**	^c^**CI**	^d^**Adap mut**
anc	Control	1	**788**	10/10	**788,1611, 563**	5/9	n, Y	5
					**788,1611, 587**	2/9		
					**788,1611**, 576	2/9		
anc	Benign	1	**788**	2/10	**1611**	2/10	Y, Y	3
			**1611**	2/10	**1611, 563**	3/10		
			878	5/10	**1611, 593**	5/10		
			878, **1702**	1/10				
anc	Harsh	1	ND		**3129**	9/9	--, n	1
anc^788^	Control	2	ND		**181**, 1033	8/10	--, y	3
					**181**, 1033, 1216	1/10		
					**181**, **1295**	1/10		
anc^788^	Control	3	none	1/10	1302	1/10	Y, Y	3
			1302	3/10	1302, **587**	6/10		
			**1702**	1/10	1302, **593**	2/10		
			3340	4/10	1302, 1690	1/10		
			3340, **581**	1/10				
anc^788^	Benign	2	none	5/9	**563**	8/10	y, y	3
			**563**	2/9	**1295**	1/10		
			**587**	1/9	**181**	1/10		
			624	1/9				
anc^788^	Benign	3	ND		**563**, 2772	8/9	--, n	2
					**563**	1/9		
anc^788^	Harsh	2	ND		**3129**, 2973	10/10	--, n	2
anc^788^	Harsh	3	none	2/9	3039	2/10	n, n	2
			3039, 1138	1/9	3039, **2971**	8/10		
			3039	6/9				

^a ^Numbers indicate nucleotide positions that are different from ancestor; rows list the mutational position(s) for a single genotype. ND = not done; Bold = parallel mutations.

^b ^Total number of genotypes over total number of isolates sequenced.

^c ^CI status of mid- and endpoint populations; y means that multiple mutually exclusive genotypes are present (i.e., A versus B, or AB versus AC, but NOT A versus AB; the latter condition is simply a selective sweep in progress, not competition between genotypes). Y = strong CI, where multiple isolates of competing mutations were observed; y = weak CI, where only one competing isolate was observed.

^d ^Number of adaptive mutations in endpoint populations as determined from criteria in Table 3. Data were used for Mann-Whitney *U*-test of H_o_: The number of adaptive mutations sampled in control/benign endpoint populations is similar to the number of adaptive mutations sampled in the harsh populations (i.e., adaptive mutations were not sampled from populations with different distributions of adaptive mutations).

**Table 3:** Mutations Identified by Genome Sequencing.

**Genome position**^a^	**Treatment**^b^	nt change		**Protein/position**^c^	**Amino acid change**^d^		**Evidence change is adaptive**^e^
181	C, B	G	T	C17, K44	A, C	S, F	Parallel High frequency Previous (3)
563	C, B	C	T	(D58)	-	-	Parallel High frequency Previous (1)
576	C	C	T	(E3), (D63)	-	-	High frequency Previous (1)
581	C	C	T	E5, (D64)	T,-	I,-	Previous (1)
587	C	G	T	E7, (D66)	W, -	L, -	Parallel High frequency
593	C, B	C	T	E9, (D68)	T, -	I, -	Parallel High frequency Previous (1)
624	B	G	T	D79, E19 E74,	A, L	S, F	**None**
788	C, B	C	T	(D133)	T,-	M,-	Parallel High frequency Measured
878	B	T	C	J11	C	N	High frequency
1033	C	G	T	F10	M	I	High frequency Previous (1)
1138	H	C	T	(F45)	-	-	**None**
1216	C	C	T	(F71)	-	-	**None**
1295	C, B	A	G	F98	N	D	Parallel Previous (6)
1302	C	C	G	F100	T	S	Parallel Previous (1)
1611	C, B	A	G	F203	H	R	Parallel High frequency Previous (4) Measured
1690	C	C	T	(F229)	-	-	**None**
1702	C, B	T	C	(F233)	-	-	Parallel Previous (1)
2772	B	T	C	(G126)	-	-	High frequency
2971	H	C	T	H14	A	V	High frequency Previous (6)
2973	H	G	A	H15	G	S	High frequency Previous (1)
3039	H	G	T	H37	V	L	High frequency
3129	H	G	T	H67	A	S	Parallel High frequency
3340	C	A	G	H137	G	D	High frequency Previous (4)

^a ^Genome position of nucleotide mutation.

^b ^C = control; B = benign; H = harsh.

^c ^Gene product (indicated by letter) and amino acid position of mutation.

^d ^Effect of the mutation at the amino acid level (from amino acid/to amino acid). Gene product C is involved in DNA replication, D is the external scaffolding protein, E is the lysis protein, F is the major capsid protein, G is the minor capsid protein, H is the pilot protein, J is the DNA binding protein, and K is of unknown function. Because φX174 has overlapping reading frames, some changes affect more than one protein.

^e ^Evidence that a particular change is adaptive includes: Parallel = occurrence in multiple lines of this study; High frequency = occurrence in multiple isolates in a population in this study, indicating the change was at high frequency in the population and that there was no evidence of hitchhiking; Previous (#) = occurrence in previous evolution experiments (the number of evolution lineages that the mutation was observed) [24,26-29, and unpublished data]; Measured = measurement of fitness effects. None = no independent evidence that the mutation is adaptive. Of the mutations considered to be adaptive by the criteria above, hitchhiking could not be ruled out for 2973 and 581. However, both of these mutations have occurred previously under very similar experimental conditions and there are additional indicators that these mutations are adaptive: 581 affects an amino acid two away from another that is adaptive in this experiment (protein E, site 7 vs E7), and is also two sites away from a site known to have a large effect on lysis regulation (E3 [32]). Mutation 2973 at H15 is adjacent to site H14 which showed strong evidence of being adaptive.

Parentheses indicate synonymous substitutions.

#### b. Number of adaptive mutations per treatment

Another parameter of the distribution of beneficial mutations that can affect levels of CI is the overall frequency of adaptive mutations that are available. If fewer adaptive mutations are available, then the waiting time is increased which could decrease levels of CI. To investigate whether there were differences in the number of mutations available under the two environmental treatments, we compared the number of adaptive mutations identified in populations from the harsh and benign environments by a Mann-Whitney *U*-test. For this analysis, we pooled data from both ancestors since only one population was examined for anc in the harsh condition, and we excluded mutations that showed no evidence of being adaptive. There were significantly more adaptive mutations in endpoint populations of the control/benign treatments relative to those in the harsh treatments (*U*_3,6 _= 17, *P *= 0.039; Tables 2 and 3).

#### c. Site of mutations

In the benign environment, adaptive mutations identified in the endpoint populations were between nucleotide positions 181 to 2772, spanning several genes, and were classified into four functional groups (Table 4). On the other hand, mutations observed in the harsh treatment were all in gene H between positions 2971 and 3129. This pattern was not absolute. The C788T change at E74 in anc^788 ^was beneficial in the harsh environment even though it did not occur there, and A3340G at H137 was at high frequency in the midpoint of control line 3, but lost at the end. Nevertheless, it appeared that changes affecting different viral functions dominated adaptation under benign and harsh treatments (Tables 3 and 4), and in the benign environment, a larger number of sites in several genes was able to contribute adaptive solutions, while in the harsh environment, solutions appeared to be more limited.

**Table 4:** Frequency of Mutations in Four Genomic Regions.

Mutation site	Protein	Putative function	Control, Benign^a^	Harsh^a^
181	C, K (DNA maturation and accessory proteins)	Burst size	2	0
563, 576, 587, 593, 788	D, E (Lysis protein and its regulatory region)	Lysis timing	10	0
1033, 1295, 1302, 1611, 2772	F, G (Capsid proteins)	Attachment, stability, assembly	7	0
2971, 2973, 3039, 3129	H (Pilot protein)	Eclipse rate: DNA ejection into host	0	5

^a ^Numbers are the frequency that mutations from a particular functional category were observed in independent evolution lines under the indicated treatments.

Only adaptive mutations (i.e., satisfying criteria in Table 3) from endpoint populations are shown.

### Frequency of CI

We evaluated the frequency of CI by determining the number of endpoint populations where CI was present or absent and comparing these frequencies between treatments by a Fisher Exact Test (see Discussion and Table 3 for criteria used to identify CI; Tables 2 and 5). Again, we pooled data from both ancestors in order to increase sample size and considered the control as a benign treatment. We found that significantly more populations showed evidence of CI in the benign environments relative to the harsh environment (Table 5).

**Table 5:** Summary of Endpoint Populations Showing Evidence of CI.

Endpoint populations	Pooled^b ^Ancestors	Fisher Exact *P *<*x*
**Benign**		
**Pops with CI**^a^	**5**	**0.048**^c^
**Pops with no CI**^a^	**1**	
**Harsh**		
**Pops with CI**^a^	**0**	
**Pops with no CI**^a^	**3**	

^a ^Pops with CI: Total number of populations in each treatment (harsh versus benign) showing evidence of CI (as determined in Table 2); Pops with no CI: Total number of populations not showing evidence of CI.

^b ^Number of endpoint populations with or without evidence of CI pooled across both ancestors.

^c ^H_o_: The frequency of populations showing CI is not higher in benign conditions.

Another pattern that would support the occurrence of CI under our experimental conditions is the observation of mutations in a midpoint population that were not seen in the endpoint population of the same line. We therefore examined variation at the midpoint of some populations where the endpoint strongly indicated CI and in line 3 of the harsh treatment where variation (but not CI) was present in the endpoint population. CI was evident in three of the four midpoint populations from control and benign treatments, and all three of these had mutations that were not seen among the endpoint isolates. In two of these cases, the lost genotypes were at very high frequency (4/10 and 5/10; Table 2). In the harsh environment, there was no evidence of CI in the midpoint population, and although there was one mutation that was not observed in the endpoint population, that mutation was a synonymous mutation with no independent evidence of being adaptive.

**Figure 2:**
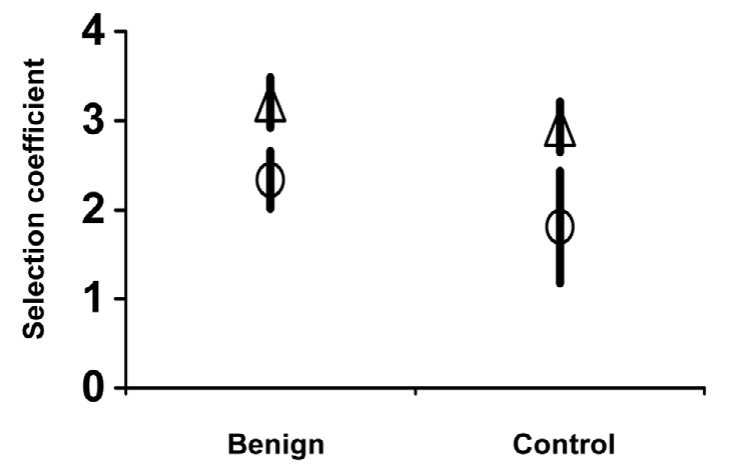
**Selection coefficients of two mutations from Line 1 control and benign treatments**. Zero indicates fitness of the ancestor. Each point is a mean estimated from five paired replicate fitness measures. Bars are standard errors of mean selection coefficients (circle: C788T; triangle: A1611G).

To examine whether mutations that were lost between the midpoint and endpoint populations could have been out-competed, we measured the selection coefficients of a mutation that showed evidence of being lost due to CI, and of the mutation that may have out-competed it (Fig. 2, Table 2). In the midpoint population of control line 1, only C788T was detected, while in the midpoint population of benign line 1 there were four genotypes, including C788T. By the endpoints, C788T was present in all genotypes of control line 1 but was not detected in benign line 1, and A1611G was detected in all genotypes in the endpoint populations of both treatments. Apparently, A1611G did not interfere with the fixation of C788T in the control line, but did interfere in the benign line. If CI were responsible for this outcome, then in the benign line, both mutations should be adaptive and A1611G should have a significant advantage over C788T. Indeed, both mutations had a significant selective advantage over their ancestor (Fig. 2; benign – A1611G: *t*_4 _= 11.7, *P *= 0.0003; C788T: *t*_4 _= 7.4, *P *= 0.0017), and the A1611G mutation had a significant selective advantage over the C788T mutation (*t*_4 _= 6.3, *P *= 0.0032), supporting that C788T was lost due to CI by A1611G in the benign line.

### Frequency of multiple mutations

Evidence supporting the multiple-mutations model versus the one-by-one model of clonal interference would be the occurrence of more than one change from the fixed genotype. We found four populations where a single isolate differed from the (putatively) fixed genotype by more than one change: benign 1 midpoint, isolate 878, **1702**; control 2 endpoint isolate 181, 1033, **1216**; control 3 midpoint, isolate 3340, **581**; and harsh 3 midpoint, isolate 3039, **1138**. (The mutation presumed to have occurred second in an already variable background is indicated in bold.) In two of these cases (1216 and 1138), the changes were synonymous in gene F and there is no independent evidence to suggest that the additional change was adaptive. In the two cases where there is evidence that the additional changes were adaptive (1702 and 581), they were seen only in the midpoint populations. Additionally, since the control lines behaved like the benign treatment, they were treated as such in the analyses.

## Discussion

Theoretical models and empirical data show that increased population sizes and mutation rates can slow the rate of adaptation and decrease the probability that beneficial mutations will reach fixation [3,12,18-22]. Phenotypic data from the empirical studies show that rates of adaptation do not increase linearly with population size, but it is not clear whether this pattern is actually due to competing mutations or simply because each mutation will have a longer transition time in larger populations. It is also not clear how many genotypes might be competing at any given time, which is an important parameter for studying properties of adaptation and predicting adaptive walks. We evolved nine lines of phage and sequenced genomes of multiple individuals from populations in these lines to examine the underlying genetics of CI. We found that mutations with relatively large selection coefficients can be out-competed and that there were as many as three competing genotypes in many of the populations where resource was abundant, even though the regular strong bottlenecks inherent to our flask-to-flask transfer protocol strongly decreased our ability to observe most mutations. This suggested both that beneficial mutations and CI were remarkably prevalent. Our sequence data did not reveal any CI in the harsh treatment, which emphasized that the frequency of CI can vary among similarly large populations due to environmental conditions.

### Adaptation under different levels of resource

The resource treatments were intended to alter parameters of the distribution of beneficial mutations in order to evaluate environmental effects on levels of CI in similarly large evolving populations. While we found evidence that there were differences between lines in the available beneficial mutations, these differences were not as we predicted. We expected that the ancestor would be further from its fitness optimum under harsh resource conditions, which would result in a larger mean selection coefficient for beneficial mutations. However, there was insufficient evidence that the mean selection coefficient of beneficial mutations was higher in the harsh environment for anc, and it even appeared to be *lower *in the harsh treatment for anc^788^. Apparently, the C788T mutation in anc increased fitness in the harsh environment (Fig. 1A), such that selection was weaker on anc^788 ^than on anc in the harsh environment. Also, although we expected that the variable resource levels would select for improvement on the same trait, the sequence data suggested that the stronger selection was on different targets between the two resource treatments, and possibly more genetic solutions were available in the benign environment. Thus, it appeared that the distribution of beneficial mutations differed in overall frequency of adaptive mutations between the resource treatments, such that more mutations were available in the benign environment.

### Factors affecting the frequency of CI

When considering the overall frequency of CI for both ancestors, we observed CI in most populations in the benign environments but not in any populations in the harsh environments (Tables 2 and 5). While this result was consistent with our original prediction, it was not attributed to a larger mean selection coefficient in the harsh environment as we had predicted. For example, selection was possibly weaker for anc^788 ^in the harsh environment, yet no CI was observed in any of those populations. Additionally, although we found that the selection coefficient of the mutation that occurred in the harsh environment of line 1 was very high, it was the only mutation observed, and thus chance cannot be out-ruled as an explanation for the difference in selection strength between the treatments in line 1. However, there was evidence of an overall trend of fewer adaptive mutations in the harsh condition (Tables 2 and 3), suggesting that the lower levels of CI in the harsh environment may have been due to the lower number of available adaptive solutions, which would increase the waiting time for beneficial mutations and decrease their co-occurrence [3,12,16,17].

Interestingly, even though the frequency of CI appeared to differ among the two resource treatments, their overall increase in fitness was similar, as were the population sizes among experimental lines. This result emphasizes two important points: 1) that CI does not necessarily decrease rates of population adaptation, and 2) that levels of CI are not necessarily predictable from population size. Regarding adaptation rate, it is indeed possible that CI acts to increase population adaptation by maintaining higher levels of genetic variation. For example, if a very high fitness peak can only be achieved through a specific ordering of substitutions, delayed fixation time from CI could increase the probability that primary mutations in the order are maintained long enough for the subsequent necessary mutations to arise. In the absence of CI, fixation of alternate mutations may be too rapid, occurring before the critical primary mutations, which could result in the population being stranded on a lower fitness peak than what could have been realized through a different adaptive walk. In this manner, the decreased rates of adaptation that are predicted to occur from competing mutations, could be outweighed by larger final fitness gains due to the more thorough exploration of sequence space that occurs in populations undergoing CI. This could partly explain our observation of similar levels of adaptation, but different levels of CI, among the two resource treatments.

There appeared to be fewer adaptive solutions available in the harsh treatments since fewer different mutations were observed and most occurred in a single gene. All substitutions in the endpoint populations of the harsh treatment were in gpH, a capsid protein involved in guiding phage DNA into host cells [33,34], while those detected in the control and benign environments occurred in several different genes, including the gene encoding the major capsid protein (gpF), as well as in genomic regions that are putatively involved in regulation of lysis timing [32]. GpF is involved in numerous processes throughout the phage life cycle including attachment to hosts, catalysis of eclipse, and assembly [35-37]. The apparently larger number of genetic solutions in the benign environment relative to in the harsh environment suggested that: 1) selection acted on a broader range of phenotypes in the benign treatments, and/or 2) there were more genetic solutions to the types of phenotypes under selection in the benign treatments. In the harsh environment where Ca^+2 ^was very limiting, there should be strong specific selection on capsid proteins to either be able to inject DNA with fewer Ca^+2 ^molecules or to become more efficient at acquiring Ca^+2 ^since genome injection is crucial to initiating infection. In other words, in the harsh environment, improvement of this trait must come before other potential renovations to growth rate. In contrast, in the benign environments where growth rates were already high at the outset, modification of any phenotype affecting growth rate (i.e., attachment, penetration, replication, assembly, lysis timing, etc.) could be effective at increasing fitness. For example, one strong source of selection inherent to all treatments is bottleneck survival. Faster lysis is a conceivable adaptive response under these conditions: phage genotypes with the highest number of free phage immediately before transfer (i.e., phage that have propagated and lysed) will have the highest probability of being transferred to the next flask, and these genotypes will go on to initiate the most individual infections in the subsequent flask. Thus, in the benign conditions adaptation could occur through any modification that produced a high number of phage by the end of each flask growth period whereas in the harsh environment selection specifically targeted injection rates before any other process in the phage life cycle, suggesting that the higher levels of CI in the benign environment could have been due to a larger number of adaptive targets under these conditions.

### Experimental design issues

#### a. Bottleneck effects

Since phage had higher growth rates in the benign environment, bottlenecks to a smaller population size were required to maintain population sizes similar to those in the harsh treatments (Table 1). Thus, beneficial mutations that arose in the benign condition had a higher probability of extinction due to bottlenecks [38], suggesting that our design was slightly biased towards finding mutations in the harsh condition. Interestingly, we found fewer adaptive mutations and lower levels of CI in the harsh treatment where the extinction probabilities of beneficial mutations due to bottlenecks were lower. This demonstrates that the difference in CI between the two treatments may have been even more dramatic (i.e., more frequent in the benign condition) if bottleneck sizes were equal. Thus, our approach to testing the hypothesis that CI would be stronger in benign environments was conservative.

#### b. Growth rate differences

The combined effect of differences in growth rates for phage in the two treatments and frequent, regular, strong bottlenecking is an experimental design factor that could have increased the extinction probability of mutations in the harsh environment. Previous work has shown that phage with lower growth rates have increased burst times [39], suggesting that replication is slower. If the higher growth rates of phage in the benign environment translate to faster replication relative to that in the harsh environment, then mutations would arise earlier in flask growth. From the work of Wahl and Gerrish [38], we found that mutations arising in the last five minutes of flask growth have a greater than 99% chance of being lost due to sampling, regardless of their selection coefficients, while those arising in the first five minutes are likely to survive the bottleneck if their selection coefficients are high. Thus, the lower growth rates of phage in the harsh environment could have increased the extinction probability of beneficial mutations if most were produced too late during flask growth to survive the bottleneck. If present, this effect could explain the lower levels of CI observed in the harsh condition. However, it is also possible that the less severe bottleneck in the harsh condition, which decreased extinction probabilities (see previous section), acted to negate potential effects from low growth rates.

### Implications of CI for predicting adaptation

In four of the five lines where we sequenced isolates from the midpoint populations, we found genotypes that were no longer present in the endpoint population, which we refer to as 'lost.' Furthermore, from measuring the selection coefficients of one of these lost individuals we found that mutations with relatively large selection coefficients can be lost. These data demonstrated two important points that are relevant to understanding and predicting adaptation. First, the probability that a beneficial mutation is lost due to a deterministic evolutionary phenomenon such as CI depends partly on stochasticity from the time that other mutations of similar effect arise. In populations that are subject to strong sampling effects, it is possible for a small-effect mutation to reach fixation in the presence of a larger one, as long as the smaller-effect mutation is near fixation before the larger-effect mutation arises, such that the larger-effect mutation is more strongly affected by the stochastic sampling. Second, the fact that relatively large-effect beneficial mutations were lost in competition supports the finding that beneficial mutations may be much more frequent than believed [40,41]. Many beneficial mutations are often not seen because they can only be sampled in environments where selection coefficients are similar among mutations, or where there are no mutations with very large selection coefficients. This emphasizes the importance in measuring adaptation in numerous environments in order to develop predictive models and understand evolutionary processes.

The co-occurrence of high frequency beneficial mutations has been previously observed during experimental evolution of microvirid phages in batch culture (φX174 and G4 phages; [23]). We found evidence for CI in five out of nine populations even though all mutations had high extinction probabilities, suggesting that CI can be remarkably common in large bacteriophage populations. While in most cases competing genotypes differed from the (putatively) fixed genotype by only by a single adaptive mutation, which supports predictions of the one-by-one model of clonal interference, we found limited support for the multiple mutation model. There were four cases where a single isolate differed from the fixed genotype by more than one change, although in two of these cases there is no evidence that the additional change is adaptive. In no case did these genotypes carrying multiple mutations dominate in the endpoint populations. Taken together, our data and the recent study in yeast emphasize that concurrent mutations are an important determinant of adaptive trajectories in large microbial populations. Nevertheless, sequence-based models of adaptation that assume selection is strong relative to mutation (Strong Selection Weak Mutation assumption, SSWM), such that one beneficial mutation is fixed before the next occurs, have been considered acceptable for describing properties of adaptive walks in asexual organisms [42-44]. Our experimental data suggest that SSWM conditions may often be unrealistic in large microbial populations, and thus that models of adaptation relying on this assumption may make misleading predictions regarding the properties of adaptive walks in large microbial populations. Models of adaptation that include concurrent mutations and multiple concurrent mutations on individual genotypes [3,9] may provide more realistic frameworks for investigating the properties of adaptation in large microbial populations.

## Conclusion

We verified with sequence data that CI occurs in large virus populations and that several genotypes may co-occur at very high frequencies. We intended to test the prediction that weak selection in benign environments would result in more CI on average. While our data showed that CI was more frequent in benign treatments, this difference was not attributed to weaker selection. It appeared that the higher frequency of CI in the control and benign environments was partly due to a higher number of potential adaptive solutions in those environments, relative to that in the harsh treatment, which would have decreased the waiting time for beneficial mutations. At the very least, our results showed that environmental conditions can alter the frequency of CI among similarly large populations, which demonstrates that effects of CI are not necessarily predictable from population size. Another important insight from our genetic data was that selection appeared to target different phenotypes in treatments where the same continuous resource variable was decreased to different degrees. Finally, we observed that the fate of mutations with relatively large selection coefficients depends on the time that other large-effect mutations arise, suggesting that these stochastic population-level processes are significant properties of adaptive landscapes, as predicted by theoretical models.

## Methods

### Biological system

φX174 is a single-stranded, lytic DNA bacteriophage. Its genome is 5386 bases and consists of 11 genes [45]. φX174 proliferates rapidly; on average a high fitness phage produces 100 mature progeny in a single lytic cycle which takes 20 minutes (generation time). A lipopolysaccharide (LPS) mutant derivative of *E. coli *F470 (CGW317), which produces rough LPS with a full inner and outer core but no O-antigens, was the host used in all evolutions and fitness assays [46].

### Experimental evolution conditions

An isolate of a lab strain of φX174 (GenBank accession AF176034) was pre-adapted to our experimental conditions: serial flask transfers in 20 ml liquid media (10 g/L tryptone, 5 g/L yeast extract, 10 g/L NaCl, and 2.0 mM CaCl_2_) with 30-minute growth periods per flask on *E. coli *CGW317. Flasks were shaken in a water bath at 200 rpm at 38.5°C. To maintain a constant, homogenous environment and to eliminate coevolution between phage and hosts, each flask was inoculated with a sample of host cells from a pre-prepared stock. The stock of hosts had been amplified as a large batch, harvested during the exponential growth phase, divided into individual tubes, and stored at -80°C. Naïve host cells were added to each flask at a density of 4 × 10^7 ^cells/ml (OD_600 _= 0.05). Flasks were then incubated for one hour such that cell density reached 1 × 10^8 ^cells/ml (OD_600 _≈ 0.13) before the addition of phage. After each 30-minute flask growth period, cultures had reached densities of about 4 × 10^8 ^cells/ml (OD_600 _≈ 0.5), which is about 8 × 10^9 ^total cells. Phage were inoculated at a multiplicity of infection of 0.005 to 0.1, depending on their growth rates. Each line was initiated from an isolated plaque (clonal population). We monitored adaptation by measuring numbers of phage at the start and end of each 30-minute flask growth period and calculating the population growth rate [39]. Samples from each flask were stored at 4°C and -20°C. We aimed to maintain phage populations at lower densities than hosts to decrease the probability of co-infection and maintain selection on growth rate. Despite these efforts, titer data from each flask during the trajectory showed that some phage populations reached the same size as bacterial populations, suggesting that co-infection could have occurred at later stages during flask growth in some of the cells. Since φX174 is thought to be capable of recombination during co-infection, this suggests that low rates of recombination were possible [47,48]. However, this was likely to be rare since phage densities only approached those of hosts late during flask growth, and each transfer involved a bottleneck to a smaller population size such that co-infected hosts would be subject to strong sampling effects.

### Experimental design

We pre-adapted the laboratory strain (above) to our experimental conditions in order to obtain a starting genotype that was very near a local fitness optimum. The pre-adapted genotype was then evolved further under different levels of CaCl_2_, a resource that is important for attachment and required for catalyzing a conformational change in phage capsid proteins, which results in injection of the phage genome into its host [35,37,49]. Our CaCl_2 _treatments were: 1) identical to pre-adaptation conditions (control), 2) slightly lower CaCl_2 _concentration with undetectable differences in phage growth rate (benign), and 3) much lower CaCl_2 _concentration with significantly lower phage growth rate (harsh). This design was expected to expose the ancestral genotype to different parameters of the distribution of available beneficial mutations by changing its proximity to a local fitness optimum. Experimental parameters for each line are shown in Table 1.

Each experimental line was evolved until the sum of phage offspring from all flasks in the line (i.e., the cumulative number of progeny produced) was equal to 6 × 10^11^. Midpoint populations were sampled at 3 × 10^11 ^total genomes. We chose total new genomes as our measure of evolutionary time instead of the usual measure, generation time, in order to allow for equal numbers of mutations in each resource treatment. In these phage, generation time is equivalent to a full lytic cycle, and burst size can be much higher for phage with high growth rates relative to those with low growth rates. Thus, the opportunity for mutations to arise, and for clonal interference to occur, would differ between phage in benign and harsh environments if we used generation time as our measure of evolutionary time. Under the simplifying assumption that all mutations are equally likely, 6 × 10^11 ^total progeny means that every possible mutation could have occurred 1.7 × 10^5 ^times according to the following rationale. The total number of genomes (6 × 10^11^) multiplied by the mutation rate in number of mutations per genome per replication (0.0046; [50]) gives the total number of mutations (2.8 × 10^9^). Since there are 5386 sites in the genome and three possible mutations per site, the number of possible mutations in the genome is 5386 × 3 = 1.6 × 10^4^. The total number of mutations that occurred (2.8 × 10^9^) divided by the total number of possible mutations (1.6 × 10^4^) gives the average number of times each possible mutation occurred at each site, which is 1.7 × 10^5^. This is a rough estimate due to transition/transversion bias, among-site rate variation, and strong bottleneck effects from the flask-to-flask transfer regime. However, it does suggest that all mutations should have arisen many times during our experiments.

### Criteria for detecting CI from sequence data

Each line was founded by a single genotype and populations underwent frequent, strong bottlenecks during their evolutionary trajectories. Under these conditions, CI can only occur after beneficial mutations arise independently and increase in frequency enough to survive regular bottlenecks (i.e., at least 0.5–10% under our experimental conditions). Since we sequenced only a small fraction of individuals (10) from a very large population (7 × 10^9^), we examined only 0.00000014% of the population on average. By this sampling scheme, rare genotypes have a low probability of being observed. Thus, we concluded that there was evidence for CI in populations that contained multiple genotypes with at least one novel mutation (i.e., AB versus AC, but not A versus AB; the latter was considered to be a selective sweep in progress). To be conservative in our method of identifying CI, we only considered mutations that showed molecular evolutionary evidence of being adaptive (criteria described in Table 3 and Discussion).

### Growth rate assays

Growth rate for each endpoint population was measured under the same conditions in which the population was evolved by previously described methods [24,39]. Five paired replicate assays of the ancestor and evolved endpoint populations were conducted for each of the nine lines (45 paired fitness assays). Briefly, phage were incubated with hosts for one hour, isolated by adding chloroform to lyse the cells, and separated from hosts by centrifuging for four minutes at 15 K rpm. Initial and final population sizes (*N*_*o *_and *N*_*t*_, respectively) were estimated by titering. Fitness assays for mutants with a single nucleotide difference relative to wildtype (i.e., C788T or C1611T) were conducted in the same manner except that, rather than using population samples, the genotypes were isolated from a single plaque and confirmed by genome sequencing.

### Estimates of fitness and selection strength

Our measure of fitness is log_2 _of the absolute growth rate (*r *= *N*_t_/*N*_o_, where *t *= 1 hour). This is equivalent to the number of population doublings per hour, and is denoted *w*. Fitness of evolved populations relative to ancestor was calculated as a ratio of the absolute growth rate of the evolved population relative to that of the ancestor. This ratio was log transformed due to the exponential property of phage growth to yield relative fitness (*relW *= log_2 _[(2^r^)_evo_/(2^r^)_anc_], which is equivalent to the 1+ *s *formula used in population genetics). We use the terminology 'selection coefficient' when referring to the single mutants, but the calculations were done in the same way as those for relative fitness. As a measure of selection strength, we estimated the mean selection coefficient (*S*) of mutations from each endpoint population as follows: [∑(*relW*_*k *_× *n*_*i*_)_*j*_]/*N*_*k*_, where *n *is the frequency of mutation *i*, *N *is the total number of unique mutations, *k *denotes the endpoint population, and *j *is the summation set from 1 to *j*. As a second measure of selection strength, we calculated the proportional contribution of increased fitness to total fitness measured at the end of the evolution trajectory (*relW*/*w*_*end*_).

### Genome sequencing

Mid- and endpoint populations were serially diluted and plated on a lawn of sensitive hosts to allow plaque formation. Ten plaque isolates were arbitrarily selected for genome sequencing (129 genomes were sequenced in total). Genome sequencing was carried out as previously described [27].

### Statistical analyses

We analyzed relative fitness and selection strength data using general linear models (ANOVA and nested ANOVA) and *t*-tests in JMP (Version 5.1, SAS Institute Inc., Cary, NC). We tested whether the number of adaptive mutations was similar between harsh and benign environments by a Mann-Whitney *U*-test (StatView 5.0.1, SAS Institute Inc., Cary, NC). We examined the frequency of clonal interference by a Fisher Exact Test [51].

## Authors' contributions

KMP carried out the experiments and analyzed the data. KMP and HAW conceived the study and wrote the manuscript. Both authors have read and approved the final version of the manuscript.
